# Analysis of risk factors affecting obesity in Korean adolescents: based on the 2017–2020 Korea national health and nutrition examination survey

**DOI:** 10.3389/fnut.2025.1554218

**Published:** 2025-04-24

**Authors:** Joon Young Kim, Kyungchul Song, Youngha Choi, Byung-Sun Choi, Hyun Wook Chae

**Affiliations:** ^1^Department of Pediatrics, Yonsei University College of Medicine, Seoul, Republic of Korea; ^2^Department of Preventive Medicine, Chung-Ang University College of Medicine, Seoul, Republic of Korea

**Keywords:** obesity, adolescent, sleep duration, sedentary time, protein intake ratio, dinner skipping

## Abstract

**Background:**

Obesity, which is caused by various congenital factors, lifestyle changes, and modernized eating habits, has recently emerged as a serious health concern in children and adolescents. According to the Korea National Health and Nutrition Examination Survey (KNHANES) conducted from 2007 to 2020, the prevalence of obesity and overweight in youth has shown an increasing trend over time. Notably, obesity has been studied in several studies. However, given its close association with rapidly changing societal environments and lifestyle patterns, continuous updates are necessary. Therefore, investigating the current state of obesity in children and adolescents is crucial. Herein, we investigated factors related to the prevalence of obesity.

**Materials and methods:**

We investigated 1912 adolescents between the ages of 12 and 18 years from 2017 to 2020 using data from the KNHANES. Known and suspected risk factors for lifestyle and eating habits were analyzed using univariate and multivariate logistic regression analyses.

**Results:**

Our study revealed a gradual increase in the proportion of individuals having overweight and obesity. Regarding risk factors for obesity, in the unadjusted model, older age (Odds ratio [OR], 1.11), sedentary time ≥12 h (OR, 1.29), and a higher calorie intake from protein (OR, 1.06) were positively associated with obesity, whereas female sex (OR, 0.52) and sleep duration ≥8 h (OR, 0.56) were negatively associated with obesity. These trends remained consistent in the adjusted model, with older age (OR, 1.10), sedentary time ≥12 h (OR, 1.46), higher protein intake (OR, 1.05), and skipping dinner ≥3 times per week (OR, 2.22) being positively associated with obesity and female sex (OR, 0.60) and sleep duration of 6–8 h (OR, 0.54) and ≥8 h (OR, 0.48) being negatively associated with obesity.

**Conclusion:**

Obesity in Korean adolescents was significantly correlated with shorter sleep duration, more sedentary time, higher protein intake, and frequent meal skipping at dinner. These results highlight the necessity of focused public health initiatives that support healthy living practices, including healthy eating habits, less sedentary activity, and more sleep time.

## 1 Introduction

Obesity is an established public health concern in many countries that is progressively increasing among children and adolescents. The global prevalence of obesity in children and adolescents increased significantly between 1975 and 2016, rising from 0.7 to 5.6% in boys and from 0.9 to 7.8% in girls. The prevalence of obesity has gradually increased, with a particularly pronounced rise observed in developing countries. Despite these regional differences, almost all countries have reported an upward trend in obesity, highlighting the urgent need for effective public health interventions (1). In a previous study in which the prevalence of obesity in children and adolescents aged 2–18 years in Korea was investigated, the prevalence of obesity in 2005 was 9.7%, representing a 1.7-fold increase than that in 1997 (2). In another study conducted in 2008 in which the prevalence of overweight and obesity among individuals aged 10–18 years between 1998 and 2008 was compared, the prevalence of overweight and obesity increased from 6.3 to 14.7% and from 4.6 to 8.2%, respectively. Notably, a significant difference was observed in male youth (3). Similarly, increasing rates of obesity and overweight in children and adolescents have been observed in many studies, and this is a common problem in both developed countries in the past and developing countries in the present (4). This has been predicted to be a complex result of various factors, such as changes in lifestyle and modernized eating habits, and is a serious situation in the general population (5, 6).

In addition to the increasing prevalence of obesity, various obesity-related complications have been reported. Obesity is associated with various diseases such as dyslipidemia, hypertension, sleep obstructive respiratory disease, asthma, gastroesophageal reflux, hypersensitivity colitis, diabetes, and depression (7). Diabetes, metabolic syndrome, and cardiovascular disease in children and adolescents become serious as they progress and worsen even in adulthood; therefore, early diagnosis and prevention are essential (8, 9). Furthermore, endocrine issues, such as polycystic ovary syndrome and central precocious puberty, and psychological problems, such as attention-deficit hyperactivity disorder, have all been reported (10, 11). There have also been studies on dental health, such as the occurrence of caries (11).

Obesity is caused by a combination of congenital factors, life environment, behavioral factors, and eating habits. As obesity and overweight can also be caused by acquired conditions, it is important to identify and prevent the risk factors that significantly affect obesity at the individual level. In previous studies that analyzed contributingfactors in children and adolescents, parental overweight or obesity history, parental history of adult diseases, eating a lot at once until full, eating unhealthy foods, intake of carbohydrate and fat, skipping meals and sedentary lifestyle, such as watching TV and using a computer for >1 h, were associated with obesity (5, 12–14). In another study that analyzed abdominal obesity and lifestyle in adolescents, a significant association was found between abdominal obesity and long video viewing time and a high percentage of fat in calorie intake (15). A more detailed examination of dietary habits reveals that an overall increase in food intake has contributed to the rising prevalence of obesity (16). Additionally, previous study have reported that the consumption of cooking oil has increased over time, reflecting broader dietary shifts associated with modernization (17). Furthermore, more recent research has shown that prolonged screen time, decreased physical activity, and increased consumption of ultra-processed foods have become more significant risk factors for obesity (18). Considering the intense educational environment in South Korea, where students frequently face high academic demands, examining the influence of sleep duration and sedentary time during adolescence is crucial (19). Beyond the commonly discussed factors mentioned above, several studies have highlighted the influence of mother’s education level and low income, which often leads to increased consumption of low-nutrient, high-calorie foods (20). Social networks during adolescence significantly influence obesity risk, with research indicating that peer obesity is associated with a higher prevalence of obesity (21). Furthermore, some studies emphasize that early-onset obesity is a critical determinant of obesity in later life (22, 23).

Investigations on the risk of pediatric obesity have been conducted in the early and mid-2000s; since then, it seems necessary to study the recent reality of obesity in children and adolescents in terms of increased sedentary lifestyle, reduced physical activity, and Westernized eating environments (1, 19). The widespread use of personal computers, particularly the proliferation of the internet and smartphones, has significantly transformed the daily lives of adolescents and the general population. These technological advancements have led to substantial changes in lifestyle habits. In this context, various factors have contributed to a global decline in physical activity among adolescents, as well as an increase in screen time (24, 25). Notably, regarding the population examined in the present study, previous research has reported that Korean adolescents tend to have shorter sleep durations than the average of the global population (26). A previous study has shown that various factors can affect obesity in children and adolescents, but these factors differ depending on the period and age group of the survey. Moreover, there is a lack of recent research reflecting the impact of ongoing societal changes on these associations.; therefore, we aimed to identify the main factors affecting adolescent obesity using recent data at the national level, with an additional focus on the impact of recent societal changes. As South Korea is known for its strong academic culture and heavy academic burden on students compared to other countries, conducting current research that takes these lifestyle factors and sociocultural context into account is both necessary and meaningful (27).

## 2 Methods

### 2.1 Subjects

This study used data from the Korea National Health and Nutrition Examination Survey for youths aged 12–18 years. We used data from 2007 to 2020 to assess trends in the population proportions of individuals with overweight and obesity and the average body mass index (BMI). We used data from 2017 to 2020 to identify the various risk factors that affect obesity in adolescents.

### 2.2 Survey methods

The Korea National Health and Nutrition Examination Survey is a statutory survey on the health behaviors of people, prevalence of chronic diseases, and actual state of food and nutritional intake conducted according to the National Health Promotion Act. This survey was conducted every 3 years from the 1st period (1998) to the 3rd period (2005), after which it was reorganized into an annual survey system and has been conducted annually until now. Although the proportion of children and adolescents among the surveyed population is not large and data on important behavioral patterns for the child and adolescent groups are insufficient because of the nature of the survey, it is a wide-ranging survey covering all regions of the country. It is judged to be suitable for understanding the changing trends of obesity in children and adolescents nationwide. The survey components include basic demographics, such as age, sex, and income; health surveys, including medical use, weight control, sleep health, physical activity, drinking, and smoking; and examination surveys, including body measurements and laboratory tests. Nutritional surveys are also included. Regarding energy intake assessment, the data utilized in the present study were obtained through a dietary recall survey, in which participants reported the types and amounts of food they consumed 2 days before the survey. The reported food items were then matched with a pre-analyzed nutrient composition database to estimate daily nutrient intake. This study was approved by the Research Ethics Review Committee of the Korea Centers for Disease Control and Prevention. Raw data from 2017 to 2020 were analyzed based on the request procedure and approval for the use of data for research purposes (28, 29).

Establishing an appropriate standard for obesity in childhood and adolescence is difficult due to the nature of the period during which the body changes and grows rapidly. In academic circles, the obesity standard presented in the ‘2007 Korean Child and Adolescent Growth Chart’ for obesity in children and adolescents is the most commonly used (30). In the 2007 Korean Child and Adolescent Growth Chart, which was developed and published based on domestic and international growth charts, obesity was defined as a BMI of the 95th percentile or higher by age or a BMI of ≥25 kg/m^2^ as an absolute value, which is the standard for adult obesity. Individuals above the 85th percentile and below the 95th percentile by age were classified as overweight (30).

For the analysis of risk factors for obesity, variables were selected using the *t*-test for continuous variables and the chi-squared test for categorical variables, and the correlation between obesity and risk factors was analyzed using multivariate logistic regression. The unadjusted model was used in the univariate analysis, and the adjusted model was used in the multivariate analysis, with candidates with a *p*-value of <0.1 in univariate analysis and previously known variables. Statistical analyses were performed using SPSS version 26. Statistical significance was confirmed when the p-value was less than 0.05.

## 3 Results

### 3.1 Trend of population proportions of individuals with overweight and obesity

**Figure 1:**
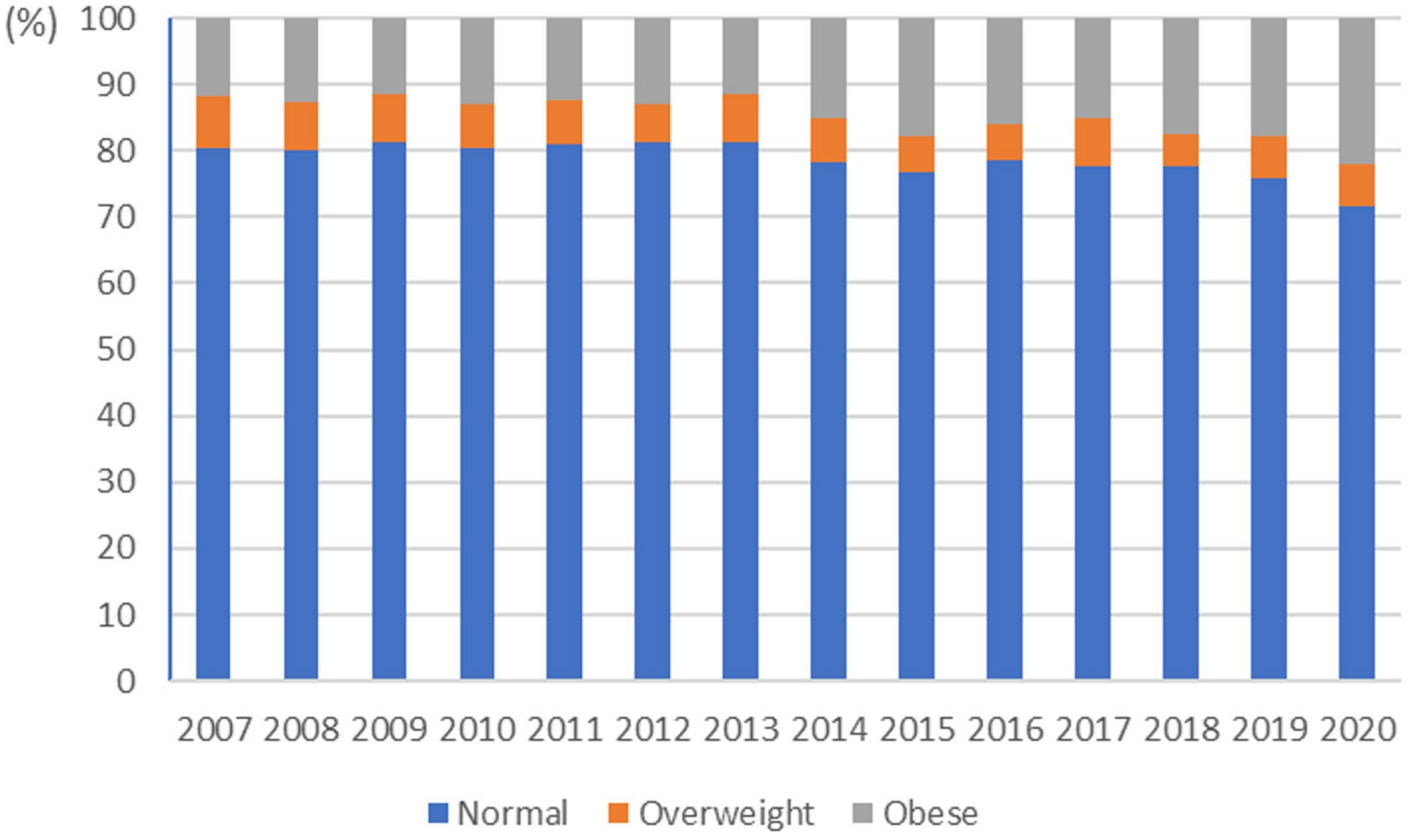
Trends in the prevalence of overweight and obesity among Korean adolescents, 2007–2020.

In this study, we investigated the trends in obesity over different periods to assess the severity of obesity prevalence. Between 2007 and 2020, adolescents aged 12–18 years were selected from the Korea National Health and Nutrition Examination Survey. The proportions of individuals with overweight and obesity were obtained (Figure 1).

### 3.2 General characteristics of the study participants

The sex distribution of the 1912 adolescents between the ages of 12 and 18 years included in this study was 53.6% males and 47.7% females, with an average age of 14.89 ± 2.04 years. Male participants were 14.89 ± 2.05 years old, and female participants were 14.89 ± 2.03 years old, with no significant difference (*p* = 0.974). The other demographic characteristics are listed in Table 1. More males drank alcohol than females (29 vs. 24%, *p* = 0.018), and more males smoked than females (12 vs. 6%, *p* < 0.001).

**Table 1:** Demographic characteristics of the study participants.

Variables	Total	Male	Female	*p*-value
Age (y) (*n* = 1912)	14.89 ± 2.04	14.89 ± 2.05	14.89 ± 2.03	0.974
Residence (*n* = 1912)				
Urban	905 (47.3%)	485 (47.4%)	420 (47.3%)	0.977
Rural	1,007 (52.7%)	539 (52.6%)	468 (52.7%)	
Household income (*n* = 1908)				
1st quartile	178 (9.3%)	90 (8.8%)	88 (9.9%)	0.617
2nd quartile	489 (25.6%)	259 (25.4%)	230 (25.9%)	
3rd quartile	634 (33.2%)	335 (32.8%)	299 (33.7%)	
4th quartile	607 (31.8%)	337 (33.0%)	270 (30.4%)	
Drinking experience (*n* = 1906)				
Yes	514 (27.0%)	298 (29.2%)	216 (24.4%)	0.018
No	1,392 (73.0%)	722 (70.8%)	670 (75.6%)	
Smoking experience (*n* = 1906)				
Yes	175 (9.2%)	123 (12.1%)	52 (5.9%)	<0.001
No	1731 (90.8%)	897 (87.9%)	834 (94.1%)	

Values are presented as % or mean±standard error.

### 3.3 Characteristics of the behavioral and eating habits of the target group

In this study, the behaviors and eating habits of the groups were observed. The average sleep time was 7.55 ± 1.26 h in the total group, 7.61 ± 1.26 h in the group having healthy weight, and 7.28 ± 1.24 h in the group having obesity (*p* < 0.001). Sedentary time was 11.22 ± 2.55 h in the total group, 11.18 ± 2.57 h in the group having healthy weight, and 11.48 ± 2.46 h in the group having obesity (*p* = 0.046). The fasting time was 12.74 ± 2.59 h in the total group, 12.69 ± 2.56 h in the group having healthy weight, and 12.95 ± 2.70 h in the group having obesity (*p* = 0.099; Table 2).

**Table 2:** Behavioral and dietary habits of the study population in 2017–2020, according to obesity status.

Variables	Total	Having healthy weight	Having Obesity	*p*-value
Sleep time (h) (*n* = 1905)	7.55 ± 1.26	7.61 ± 1.26	7.28 ± 1.24	<0.001
Sedentary time (h) (*n* = 1901)	11.22 ± 2.55	11.18 ± 2.57	11.48 ± 2.46	0.046
Fasting time (h) (*n* = 1860)	12.74 ± 2.59	12.69 ± 2.56	12.95 ± 2.70	0.099
Physical activity for ≥60 min per week (%) (*n* = 1902)				
0–2 days	1,343 (70.6%)	1,122 (71.9%)	221 (71.9%)	0.009
≥3 days	559 (29.4%)	439 (28.1%)	120 (35.2%)	
Number of days of muscular strength training per week (*n* = 1906)				
0–2 days	1,381 (72.5%)	1,144 (73.1%)	237 (69.3%)	0.149
≥3 days	525 (27.5%)	420 (26.9%)	105 (30.7%)	
Daily energy intake (kcal)	2,102 ± 886.4	2,117 ± 895.1	2036 ± 845.5	0.165
Proportion of people who ate more than recommended calories (*n* = 1,549)				
	558 (36.0%)	468 (37.0%)	90 (31.8)	0.102
% of energy from micronutrients (%) (*n* = 1,549)				
Carbohydrate	59.26 ± 10.07	59.36 ± 10.06	58.85 ± 10.12	0.441
Protein	14.78 ± 4.10	14.6 ± 3.89	15.59 ± 4.86	<0.001
Fat	24.88 ± 8.60	24.96 ± 8.60	24.49 ± 8.63	0.41
Frequency of eating out per week (%) (*n* = 1,549)				
Less than once a day	668 (43.1%)	550 (43.4%)	118 (41.7%)	0.591
More than once a day	881 (56.9%)	716 (56.6%)	165 (58.3%)	
Frequency of skipping meals per week (*n* = 1,549)				
Breakfast				
0–2 days	854 (55.1%)	714 (56.4%)	140 (49.5%)	0.034
≥3 days	695 (44.9%)	552 (43.6%)	143 (50.5%)	
Lunch				
0–2 days	1,484 (95.8%)	1,215 (96.0%)	269 (95.1%)	0.486
≥3 days	65 (4.2%)	51 (4.0%)	14 (4.9%)	
Dinner				
0–2 days	1,442 (93.1%)	1,186 (93.7%)	256 (90.5%)	0.053
≥3 days	107 (6.9%)	80 (6.3%)	27 (9.5%)	
Eating meals with someone (%)				
Breakfast (*n* = 1,075)	691 (64.3%)	581 (65.7%)	110 (57.6%)	0.033
Lunch (*n* = 1,539)	1,474 (95.8%)	1,206 (95.9%)	268 (95.4%)	0.710
Dinner (*n* = 1,527)	1,295 (84.8%)	1,065 (85.1%)	230 (83.3%)	0.451

Values are presented as % or mean±standard error.

The highest proportion of indiviuals who performed >60 min of physical activity belonged to the group having obesity (35.2%). There was a significant difference in the rate of physical activity for > 3 days between the total group, group having healthy weight, and group having obesity (*p* = 0.009). Likewise, proportion of individuals who performed strength training for >3 days was highest in group having obesity (30.7%). However, there was no significant difference in the rate of strength traing for > 3 days between the total group, group having healthy weight, and group having obesity (*p* = 0.149; Table 2).

Average daily energy consumption was 2,102 ± 886.4 kcal in the total group, 2,117 ± 895.1 kcal in the group having healthy weight, and 2036 ± 845.5 kcal in the group having obesity (*p* = 0.165). The proportion of participants who consumed more than the recommended calorie intake according to age and sex was highest in the group having healthy weight (37.0%) with no significant difference (*p* = 0.102). The % of energy from each micronutrients was analyzed respectively, only the % of energy from protein showed a significant correlation (carbohydrates, *p* = 0.441; protein, *p* < 0.001; fat, *p* = 0.410; Table 2).

When the frequency of eating out per week was divided by once a day, 56.9% in the total group, 56.6% in the group having healthy weight, and 58.3% in the group having obesity ate out more than once a day (*p* = 0.591). The frequency of skipping each meal over a three-day week was analyzed separately for the group having healthy weight, the group having obesity, and the total group. Only breakfast showed a significant difference among the groups (breakfast, *p* = 0.034; lunch, *p* = 0.486; dinner, *p* = 0.053; Table 2).

### 3.4 Risk factors associated with adolescent obesity

The various characteristics presented in Table 2 were analyzed for the incidence of obesity. Among them, the variables that seemed to be related were sleep time, sedentary time, protein ratio in total calorie intake, number of days exercised for >60 min per week, number of days skipped breakfast and dinner per week, and whether breakfast was consumed. In addition, age, sex, and previously known factors, such as the amount of carbohydrate intake, frequency of exercise, household income, frequency of skipping breakfast, and frequency of eating out, were added and adjusted (5, 14, 31, 32). The multivariate logistic regression analysis showed a correlation between age, sex, average sleep time, sedentary time, protein ratio in total calorie intake, and number of days of skipping dinner during the week.

In the unadjusted model, age was positively correlated with the prevalence of obesity, with an odds ratio of 1.11 (*p* < 0.001). Male participants were 1.93 times more likely to be obese than female participants (*p* < 0.001). The prevalence of obesity was 1.78 times lower in the group sleeping for >8 h than in the group sleeping for <6 h (*p* = 0.003) per night, and the prevalence of obesity was 1.29 times higher in the group with >12 h of sedentary time than in the group with <12 h of sedentary time (*p* = 0.031). The protein intake rate as a continuous variable showed a positive correlation of 1.06 (*p* < 0.001; Table 3).

**Table 3:** Prevalence of obesity according to demographics and habits using multivariate logistic regression analysis.

Obesity prevalence	Unadjusted model^a^		Adjusted model^b^	
	OR (95% CI)	*p*-value	OR (95% CI)	*p*-value
Age	1.11 (1.05–1.18)	<0.001	1.10 (1.00–1.21)	0.040
Sex				
Male	1.00		1.00	
Female	0.52 (0.41–0.66)	<0.001	0.60 (0.43–0.85)	0.004
Average sleep time (h)				
<6	1.00		1.00	
6–8	0.71 (0.50–1.03)	0.067	0.54 (0.32–0.93)	0.024
≥8	0.56 (0.38–0.82)	0.003	0.48 (0.26–0.89)	0.019
Sedentary time (h)				
<12	1.00		1.00	
≥12	1.29 (1.02–1.64)	0.031	1.46 (1.04–2.05)	0.030
% of energy from protein				
	1.06 (1.03–1.09)	<0.001	1.05 (1.01–1.09)	0.018
Frequency of skipping dinner per week				
0–2 days	1.00		1.00	
≥3 days	1.56 (0.99–2.47)	0.055	2.22 (1.18–4.18)	0.014

OR, odds ratio; CI, confidence interval.

a Unadjusted model was analyzed unadjusted.

b Adjusted model was analyzed for age, sex, average sleep time, sedentary time, protein ratio in total calorie intake, number of days of exercise for >60 min per week, number of days of skipped breakfast and dinner per week, whether breakfast was present, amount of carbohydrate intake, household income, and frequency of eating out.

In the adjusted model, age was positively correlated with the prevalence of obesity, with an odds ratio of 1.10 (*p* = 0.04). Male participants were 1.66 times more likely to be obese than female participants (*p* = 0.004). The prevalence of obesity was 1.85 times lower in the group sleeping for 6–8 h (*p* = 0.024) and 2.08 times lower in the group sleeping for >8 h than in the group sleeping for <6 h (*p* = 0.019). The prevalence of obesity was 1.46 times higher in the group with >12 h of sedentary time than in the group with <12 h of sedentary time (*p* = 0.03). The protein ratio also showed a positive correlation of 1.05 (*p* = 0.018). The prevalence of dinner skipping was 2.22 times higher in the group skipping ≥3 days than in the group skipping <2 days a week (*p* = 0.014; Table 3).

## 4 Discussion

Overall, in this study, we established the following results: the lower the sleep time, higher the sedentary time, higher the proportion of protein intake, and more frequent the dinner skipping, the higher the prevalence of obesity. Consistent with prior research, we found that shorter sleep duration and increased sedentary time were significantly associated with a higher prevalence of obesity. However, the main differences identified in this updated analysis include the observation that proportion of protein intake exhibited an apparent association with obesity, and that skipping dinner had a significant association with obesity.

The relationship between reduced sleep time and obesity has also been reported in other domestic and international studies (33–35). Short sleep duration has a positive relationship with leptin and a negative relationship with ghrelin, and these hormonal effects may affect BMI and body weight (36). Sleep restriction has also been hypothesized to lead to excessive nutritional intake. An observational study found that adolescents who had a 5-day sleep restriction ate increased amounts of dessert (37). In another study, calorie intake increased after sleep restriction in school-age children (38). Another potential influencing factor could be the changes in eating patterns. Among Korean teenagers, the frequency of eating three meals per day has decreased, whereas the frequency of eating snacks has increased (39). It is also possible that obesity is related to increased snacking and late meal timing owing to changes in eating patterns as a result of private education, which is a representative social problem in Korea. Conversely, a decrease in sleep duration due to sleep disorders caused by obesity may also be considered (40).

Increased sedentary time is associated with obesity and metabolic diseases (41). Recently, a relationship between screen time during sedentary hours and obesity has emerged (42). However, the recent Korea National Health and Nutrition Examination Survey data did not include this item; as such, it was excluded. As mentioned above, many studies recommend efforts to reduce the risks and screen time in children and adolescents and to increase physical activity.

In the present study, no significant association was observed between physical activity and obesity in adolescents. The relationship between physical activity and obesity has been extensively analyzed across multiple studies, including meta-analyses; however, although the impact of physical activity on BMI reduction has shown variability in strength and significance, the majority consensus supports its preventive effects. Notably, physical activity has demonstrated a positive impact on metabolic parameters, including improvements in insulin resistance, triglyceride levels, and blood pressure (18). According to one study, the neighborhood environment previously had a substantial impact on physical activity among children and adolescents. However, with the increasing prevalence of indoor activities and shifts in play culture favoring screen-based entertainment, the association between neighborhood environment and physical activity has been diminishing (43).

In addition to the aforementioned factors, recent environmental and lifestyle habits associated with obesity include screen time, including TV viewing and computer gaming. The widespread adoption of smartphones has significantly influenced changes in physical activity patterns, making it a key factor that may contribute to the recent rise in the prevalence of obesity (44, 45). Excessive screen time has been widely recognized as a factor contributing to childhood obesity (46). Prolonged screen exposure, reduced physical activity, increased sedentary behavior, and greater snack consumption contribute to obesity. Moreover, a study suggested that excessive smartphone use may also contribute to a higher risk of obesity in children and adolescents (47). As mentioned earlier, in the present study, sedentary time showed a positive association with the development of obsesity, whereas sleep time showed a negative association. These findings suggest that screen time and smartphone use are associated with sedentary and sleep time, making them risk factors for obesity. More recent studies indicate that technological advancements have not only contributed to sedentary behavior but also disrupted the sleep–wake cycle, led to poor sleeping habits, and altered eating patterns (48).

A large supply of sugar-sweetened beverages and highly refined carbohydrates has become increasingly prevalent. Studies investigating these dietary components have shown that both are associated not only with obesity but also with an increased risk of type 2 diabetes mellitus (45, 49). In the present study, information on sugary beverages and highly refined foods was not collected for the studied age group. As an alternative, we included the proportion of carbohydrate intake within total daily energy consumption in our analysis. However, we did not find a significant association with this variable. One study has reported an increase in carbohydrate intake, particularly diets high in glycemic index foods, which may contribute to hyperinsulinemia-induced excessive weight gain (5). Furthermore, from the perspective of stress-motivated eating behavior, adolescents are particularly vulnerable to engaging in unhealthy eating behaviors, especially in emotionally salient contexts. This vulnerability may be attributed to maturational changes in brain regions involved in reward-seeking, which drive the consumption of highly palatable foods (50).

An increase in protein intake is likely related to an increase in BMI, and is primarily related to animal protein intake in early childhood (23). In addition, one study reported that diets rich in protein can induce the production of harmful intestinal metabolites by the gut microbiome, which may in turn exacerbate metabolic syndrome (51). In contrast, other study have reported an inverse association between protein intake and abdominal obesity, indicating a potential protective effect (52). Moreover, studies conducted in adults have suggested that a high-protein diet may be beneficial for obesity treatment and weight management (53). In recent years, there has been increasing reliance on and excessive consumption of protein, often perceived as a crucial dietary component for health. While no established data exist specifically for adolescents, a survey of Korean male university students in their 20s, whose age difference from adolescents is relatively small, considering their behavioral patterns comparable, reported that 47.5% currently use protein supplements, and 52% have used them in the past (54). Although excessive animal protein intake has been linked to an increased risk of obesity, adequate protein consumption remains essential for proper growth during adolescence. Therefore, nutritional balance should be carefully considered in adolescents to ensure optimal growth (55). Reducing animal protein intake and increasing vegetable protein intake can effectively prevent obesity (56). Unlike previous studies, which were related to the amount of protein intake, in this study, we evaluated the percentage of protein in the total calorie intake.

There was a study that reported similar findings, indicating that the association between frequent skipping of dinner and obesity has been observed (57). One of the hypotheses for this is that the group that does not eat dinner eventually consumes more calories because of appetite upregulation (58). Another hypothesis is that skipping meals in the evening is primarily related to poor dietary quality, which may be associated with obesity (59). In other words, the frequency of intake of high-calorie, low-nutritional foods such as fast foods can increase. Meal skipping has been studied in relation to obesity, yielding varied results across different studies. However, the majority of findings, including those from systematic reviews and meta-analyses, have emphasized the significance of breakfast skipping as a key factor influencing obesity risk (13, 14, 43). Considering the sociocultural background of South Korean adolescents, where academic workload is intense and after-school study sessions are prevalent, it is plausible that dinner skipping may lead to increased snack consumption and late-night eating habits (19). These behaviors could contribute to metabolic dysregulation and, in turn, influence obesity risk (39). An organized study is required in the future in this regard.

A study based on the National Health and Nutrition Examination Survey in a representative high-income country revealed associations between obesity and reduced physical activity, high protein intake, caffeine consumption, and exposure to secondhand smoke (60). In addition, population-based studies in Europe reported a strong association between increased media use and obesity during adolescence, whereas negative correlations were observed for sleep duration and participation in sports clubs (61). Review research conducted in South Asia showed that decreased physical activity, increased media use, and higher consumption of processed and high-sugar foods were associated with a higher risk of obesity (62). Meanwhile, studies in Africa did not show a clear association between lifestyle factors and obesity; however, they uniquely observed the coexistence of both obesity and underweight, with the prevalence of obesity decreasing as age increased (63). As reported in these studies, risk factors for obesity vary by region. South Korea, currently transitioning from a developing to a developed country, exhibited patterns similar to those of the US, Europe, and other Asian countries. The present study provides meaningful insights into the increasing prevalence of childhood obesity in South Korea, a country that has traditionally exhibited relatively low obesity rates than Western nations. Therefore, special attention is required to prevent the rapid deterioration of dietary and lifestyle habits due to the increasing adoption of Westernized behaviors (45).

Our study has some limitations. In this study, the factors associated with the prevalence of obesity were sleep time, sedentary time, protein ratio in total calorie intake, and frequency of skipped dinners. However, as there was no information on screen time or consumption of sugar-sweetened beverages, the relationship could not be revealed. In addition, the results were contrary to well-known research content. The number of days of exercise is a representative indicator that is inversely correlated with obesity, and the World Health Organization recommends at least 60 min of physical activity per day and at least 3 days of vigorous exercise for adolescents (64). In the present study, univariate analysis showed that the group that exercised for >3 days tended to have increased obesity. This was a limitation of this cross-sectional study. It may seem that a high number of individuals exercised because of the perception of obesity and body shape. However, only a few adolescents exercised often. In addition, as in adults, calculating moderate-intensity exercise, high-intensity exercise, and walking time, such as the metabolic equivalent of task, can reveal an accurate causal relationship with physical activity. However, such a detailed investigation was not conducted in adolescents in our study. Total calorie intake also showed a similar pattern. In addition, it should be recognized that most lifestyle items of children and adolescents may differ from the actual ones because they depend on the questionnaire. Among the data supervised by the Korea Centers for Disease Control and Prevention, the Youth Health Behavior Survey provides valuable insights into adolescents’ lifestyles. However, as this study primarily focused on the prevalence of obesity, the data from the Korea National Health and Nutrition Examination Survey, which includes directly measured height and weight, were deemed more appropriate. This study has a strength. A multivariate analysis approach was employed to assess a wide range of variables affecting childhood obesity using a large-scale, nationally representative dataset. By incorporating multiple socioeconomic, behavioral, and dietary factors, our study offers a more comprehensive understanding of risk factors for obesity compared with previous research. This comprehensive analytical approach strengthens the reliability of our findings and offers practical insights.

## 5 Conclusion

Obesity is a pre-existing social problem known to cause various complications. Recently, the prevalence of obesity in children and adolescents has been increasing, which could result in long-term complications and sequelae even after adulthood; therefore, active prevention and treatment are needed. Considering recent societal changes, we examined factors influencing the prevalence of obesity in adolescents aged 12–18 years in the present study. Decreased sleep time, increased sedentary time, increased number of evening meals skipped, and increased proportion of protein in total calories were found to be key factors influencing the prevalence of obesity. In accordance with previous research, reduced sleep duration and prolonged sedentary time were identified as key contributors to obesity prevalence, while our updated analysis revealed that proportion of protein intake showed an association with obesity, and that skipping dinner had a significant impact on obesity risk. We investigated obesity using national open data; however, detailed and structural studies are required to conduct further in-depth research. Given the evolving lifestyle patterns among adolescents, careful management and targeted interventions are essential to mitigate obesity-related health risks and promote healthier behaviors.

## Data Availability

The datasets presented in this study can be found in online repositories. The names of the repository/repositories and accession number(s) can be found in the article/supplementary material.
